# Coping with Adversity: Resilience Dynamics of Livestock Farmers in Two Agroecological Zones of Ghana

**DOI:** 10.3390/ijerph18179008

**Published:** 2021-08-26

**Authors:** Francis Sena Nuvey, Adolphina Addo-Lartey, Priscillia Awo Nortey, Kennedy Kwasi Addo, Bassirou Bonfoh

**Affiliations:** 1 School of Public Health, University of Ghana, Accra P.O. Box LG 13, Ghana; aaddo.lartey@gmail.com (A.A.-L.); panortey@gmail.com (P.A.N.); 2 Ghana Health Service, Ministry of Health, Accra P.O. Box M 44, Ghana; 3 Noguchi Memorial Institute for Medical Research, Accra P.O. Box LG 581, Ghana; kaddo@noguchi.ug.edu.gh; 4 Centre Suisse de Recherches Scientifiques en Côte d’Ivoire, Abidjan BP 1303, Côte d’Ivoire; bassirou.bonfoh@csrs.ci; 5 Swiss Tropical and Public Health Institute, Socinstrasse 57, 4051 Basel, Switzerland; 6 Faculty of Medicine, University of Basel, Klingelbergstrasse 61, 4056 Basel, Switzerland

**Keywords:** resilience, livestock farmers, vulnerability, food security

## Abstract

Despite the increasing occurrence of adverse events including droughts and conflicts, livestock farmers in Ghana continue to raise animals to support their livelihoods and the national economy. We assessed the resilience of cattle farmers (CF) to adverse events they faced using a cross-sectional survey of 287 CF in two agroecological zones in Ghana. Resilience to adversities was assessed using the Resilience Scale (RS-14). Resilience scores and categories were computed and factors that explained variations in resilience categories assessed. The farmers kept, on average, 31 cattle per household, with a majority (91%) also growing crops. Key adverse events confronting them in both districts were animal disease outbreaks, pasture shortages, and theft, with 85% (240/287) losing, on average, seven cattle (15% of the herd size) over a one-year period. The mean resilience score was 71 (SD = 8) out of 98; 52% were highly resilient. Resilience was higher in the southern district (72 versus 70), albeit not statistically significant (*p* = 0.06). The resilience significantly improved with age, each unit increase in cattle in the herd, and having experience raising livestock (*p* < 0.001). The CF have relatively high resilience to adverse events affecting their productivity. The findings provide relevant information for implementing mitigation measures to improve production by reducing animal mortalities through high-quality veterinary services.

## 1. Background

The food insecurity levels in many African countries continue to worsen over the years despite the increasing levels of agricultural entrepreneurship, which for most is crucial to achieving self-sustainability on the continent [1]. According to the Food and Agricultural Organization (FAO), Africa and South America continue to record high levels of hunger and undernourishment in their populations, leading to food insecurity. Data presented in an FAO report on African food security show that while the number of food insecure people increased between 2000 and 2018, the prevalence (%) declined from about 25% in 2000 to under 20% in 2014 and thereafter increased slightly to about 20% in 2018 [2]. The major driver of this food insecurity, according to the FAO, is climate change, which has affected the availability, access, utilization, and stability of food in the region [3]. The negative effects of climate change are leading to poor rainfall and drought events increasing in intensity and frequency and affecting the productivity of farmers [4].

Evidence about the risks and threats to livestock farming in Africa exists. Adverse events including droughts, conflicts, animal disease epidemics, and livestock theft are widely reported as vulnerabilities contributing to the productivity shortfalls in the livestock sector [5,6,7]. The occurrence of these adverse events leads to consequential losses of livestock and disturbs the livelihood system of livestock farmers [8]. Aside from the threats related to climate variability, other factors, including the high cost of farm inputs, poor communication, water shortages, and a poor market for livestock products, are also barriers constraining livestock farmers’ productivity [9,10]. However, these vulnerabilities are often studied in isolation without also looking at the individual’s capacities to overcome them [11]. This capacity to overcome adversity is referred to as resilience.

Obrist and colleagues [11] argue that vulnerability and resilience concepts need to be combined to comprehensively understand the responses of individuals to adversity. Thus, a key consideration must be an assessment of the vulnerability to which the resilience is dependent upon. Resilience, as a concept, is considered as a positive adaptation to a threat or adverse event in a manner where individuals can restructure their social circumstances and capital to react to adversities they experience daily as well as creating options that anticipate threats, the better to deal with them. This capacity can be explored at individual, community, and national levels [11]. To attain sustainability, livestock farmers should be able to successfully cope with, adapt to, and/or recover from the effects of adverse events [12]. However, the capacity to bounce back following adverse events is often intractable and requires several positive experiences before recovery is possible [13].

Livestock farmers adopt certain adaptive and coping strategies to deal with the adverse events experienced. Many farmers engage on their own in other economic activities, including raising mixed livestock species, growing crops, making handicrafts, producing charcoal, and selling livestock, to support themselves against adversities associated with livestock farming [14,15]. However, some farmers also adopt certain adaptive strategies that could be detrimental to public health. In Ghana, a recent study found that farmers purchased veterinary medicines without professional advice and sold diseased animals as a strategy to cope with increased levels of infectious animal disease outbreaks [7]. Therefore, to enhance the resilience of livestock farmers, factors such as the provision of secure food sources and markets, effective local empowerment by governments, and improving access to extension services are essential [12,16]. The application of One Health techniques in previous studies has shown incremental benefits in improving the health of people, animals, and their shared environments [17]. The concept of One Health is defined by the requirement that programmes, policies, legislation, and research are designed and implemented through collaboration between multiple sectors including human, animal, and environmental sectors in order to achieve better public health outcomes [18,19]. Livestock farmers are uniquely placed in this interconnected network. Their livelihoods and wellbeing depend on the health of their animals as well as their shared environment [20]. In the face of adversities affecting livestock production, particularly in developing countries, farmers’ resilience to these adverse events is key to their adoption of sustainable production strategies. By assessing the resilience of farmers to adversities in livestock production, we can inform policy interventions that sustainably address human, animal, and environmental problems.

Obrist and colleagues [21] posit that in order to achieve equity and improved access to health services, quality institutional policies and processes governing service delivery as well as a consideration of the individual service user’s livelihood assets and vulnerability context are critical. According to them, livelihood assets may take the form of human, social, natural, physical, or financial capital. The availability of these livelihood assets to farmers is influenced by factors, including the economy, adverse events experienced, and technology, representing the vulnerability context within which each farmer lives [21]. In applying this framework to the access to animal health services by livestock-dependent populations in Ghana, this study sought to assess the resilience levels of livestock farmers to ascertain the viability of any proposed support interventions to farmers in improving farmers’ livestock productivity and profitability. We focused on the household-level resilience by accessing the vulnerabilities livestock farmers face and their capacity to overcome these vulnerabilities in Ghana. Understanding the vulnerabilities, resilience levels, and strategies adopted by farmers is essential to help align future interventions better with the needs of farmers and the available resources they can mobilize to access these services. It is only when animal health services account for the vulnerability context of livestock farmers that service utilization can be improved to achieve the desired food safety and food security in resource-poor settings like Ghana.

## 2. Materials and Methods

### 2.1. Description of Study Site

This study was conducted in the Bunkpurugu-Yunyoo (BY) and the Kwahu Afram Plains South (KAPS) districts in the north-east and eastern regions of Ghana, in the northern and southern belts, respectively. These districts are primarily agrarian settlements with at least 80% of their respective populations engaged in agricultural activities. Livestock farming is mainly done on a free-range basis involving the rearing of cattle, sheep, goats, and pigs in both districts [22,23]. The BY district is arid; average monthly temperatures range between 16 °C and 41 °C, with a single rainfall season annually (the highest precipitation in the rainfall season was 275 mm). The KAPS district is humid; average monthly temperatures range between 20 °C and 37 °C, with two main rainfall seasons (the highest precipitation in the minor season was 372 mm 468 mm in the major season) [24].

### 2.2. Study Design

The study was a quantitative study employing a cross-sectional design with data collection done between July and September 2018. This cross-sectional survey assessed self-reported vulnerabilities, resilience, and adaptive strategies of cattle farmers in two representative districts of the northern and southern belts of Ghana. This allowed for a determination of resilience levels in this vulnerable population, while allowing for comparability of vulnerabilities and adaptive strategies among holders of similarly high-value assets (cattle) in different agro-ecological zones in Ghana.

### 2.3. Sampling

We estimated that 30% of cattle farmers in Ghana would be highly resilient within a 5% acceptable margin of error. A study among Turkana pastoral households found 29% of households were less vulnerable to climate-induced adversity [25]. Using Epi-Info version 7.2 to determine the sample size, the minimum sample required was 324 cattle farmers, with 162 farmers per district. We obtained a sampling frame of communities with cattle farmers from the veterinary service department in each district (Table S1, Supplementary Material). Twelve and ten communities were randomly drawn from a list of 55 and 40 communities in the BY and KAPS districts, respectively. Community leaders provided a list of cattle farmers, who were recruited consecutively with an average of 12 farmers per community. In the study communities, we reached 318 farmers. Of these, 287 consented to participate in the study, yielding a response rate of 90%. The inability to achieve the minimum sample did not significantly affect the desired precision (margin of error) as only 0.3% precision was lost as a result. Overall, 145 and 142 cattle farmers were recruited from the BY and KAPS districts, respectively.

### 2.4. Data Collection and Analysis

Data was collected through the administration of a structured questionnaire to the heads of 287 cattle farming households. The questionnaire was translated to three local languages predominantly spoken in the study districts: Akan, Ewe, and Bimoba, using the back-translation approach [26]. Questionnaires were administered in the respondent’s preferred local language or in English. We considered the farming household to be pastoral if they only rear cattle without also growing crops. The household was agro-pastoral if they combine cattle rearing and growing crops. We collected data on farm size, farmer type (pastoral or agro-pastoral), adverse events (vulnerabilities) affecting cattle farming, level of animal losses, support interventions available to farmers to deal with adversities, and some demographic characteristics of farmers. The resilience scale (RS-14) was used to assess the resilience of the farmers. The RS-14 is a 14-item tool that directly measures five resilience facets: self-reliance, purpose, equanimity, perseverance, and authenticity on a 7-point Likert scale, with higher scores representing strong resilience [27]; see Table 1 for a copy of the scale and the resilience characteristics assessed. The RS-14 has been previously applied in large studies of different middle-aged and adult groups, in both clinical and community settings, and among healthy individuals as well as persons experiencing symptoms of depression. In Africa, the psychometric properties of the tool was assessed among clinical students in Nigeria. The tool has shown high validity and reliability (*α* > 0.8) [28]. Cronbach’s alpha (*α*) values between 0.70 and 0.95 have been deemed acceptable measures for the validity and reliability of instruments [29]. Therefore, this tool could be particularly valuable in evaluating resilience levels among vulnerable livestock-dependent populations, especially as livestock farmers in Ghana were recently reported to have high levels of depression, stress, and anxiety compared to the general population [7].

The STATA software (version 15.1) was used in coding and analyzing the data. Descriptive analysis of the data was expressed as frequencies and proportions for categorical data and means with standard deviations for continuous data. The RS-14 Likert scale scores ranging between 1 and 7, with 1 meaning strong disagreement with stated resilient facets and 7 meaning strong agreement with stated resilient facets, were summed up to obtain resilience scores (range = 14–98) according to standard guidelines [27]. We used the median split approach [30] to categorize resilience into high (greater than the median resilience score) and low (less than median resilience score). Inferential analysis was done to identify the factors that explained variations in the resilience categories using general and generalized linear models. To allow for ease of reporting results, we used logistic regression to present the odds of a farmer having high resilience after assessing for and finding no evidence of multicollinearity among the covariates [30].

## 3. Results

### 3.1. Descriptive Results

The average age of a cattle farmer was 47 (SD = 11) years with about 93% (268/287) being male. Although 46% (132/287) of the farmers had some basic education, more than 30% (96/287) had no formal education. The majority of the farmers (92%) were married (265/287) with an average of nine dependents, and the average household size was 10 (SD = 5) persons. About 70% (200/287) of the farmers grew up in households where livestock were raised during their childhood.

The median number of cattle kept per farmer was 31 (range = 11 to 400) cattle. Additionally, about 90% (261/287) of the farmers were agro-pastoralists. The crops grown were cereals (maize, millet, and rice), legumes (beans and groundnuts), vegetables, and root tubers (cassava and yam). All the farmers in the BY district grew at least one crop. We could not assess the size of the crop farms and other household assets like land, houses, bicycles, and televisions, among others, in this survey.

The cattle farmers reported experiencing an average of eight adverse events over a five-year recall period (range 1 to 11 adverse events). All the farmers experienced at least one adverse event, which included animal disease epidemics, animal theft, pasture and water shortages, conflict with other land users, poisoning of animals, poor market prices for animals on sale, attack by other animals, and bush fires. The farmers lost on average about 15% of their cattle herds each year to adverse events, with about 85% (240/287) of the farmers affected. The major adverse events associated with cattle losses were animal diseases including contagious bovine pleuropneumonia, foot and mouth disease and brucellosis, theft of animals, pasture shortages, and conflicts with other land users.

**Figure 1:**
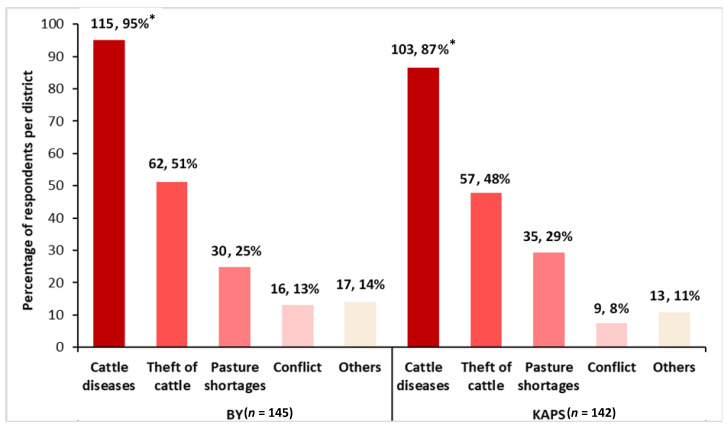
Sources of livestock losses to cattle farmers in the Bunkpurugu-Yunyoo (BY) and Kwahu Afram Plains South (KAPS) districts, Ghana. The number (*n*) and percentage (%) denote multiple responses of farmers to each source that they lost cattle to per study district (BY = 145 farmers, KAPS = 142 farmers) and are shown (*n*, %) per bar in the figure. * denotes a significant difference at the 5% level between the proportions of farmers reporting a loss to the same factor between the two districts.

As shown in Figure 1, both study districts have similarities in the major vulnerabilities they faced, as losses were mainly to cattle diseases, theft, pasture shortages, and conflict with other land users in both districts. However, the proportion of cattle lost by farmers in the KAPS district on average to animal disease outbreaks were significantly higher than the proportion lost by farmers in the BY district (12% versus 6%), *p* < 0.001. Similarly, the proportion of cattle lost to conflicts with other land users was also significantly higher in the KAPS district compared to the BY district (1.2% versus 0.3%), *p* = 0.016. The proportion of cattle herds lost to pasture shortages and cattle theft did not significantly differ across the study districts.

**Figure 2:**
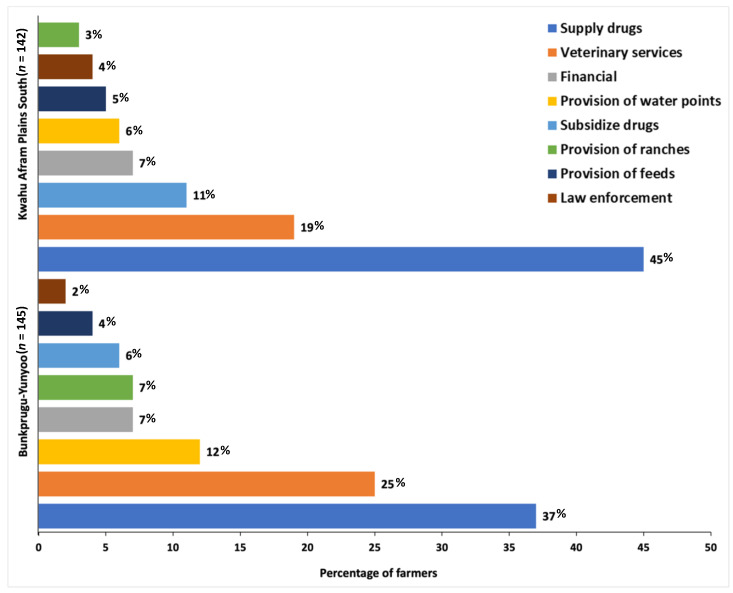
Singular priority support interventions required by cattle farmers (N = 287) to reduce losses. The percentages shown per bar were computed per study district: Bunkpurugu-Yunyoo (*n* = 145) and Kwahu Afram Plains South (*n* = 142).

The cattle farmers reported receiving support interventions on average from two main sources (range 0 to 6 support interventions), namely, veterinary service support (60% of the farmers) and support from family members (53%). Other support interventions were received from friends (39%) and livestock farmer associations (34%) in the study districts. The cattle farmers were then asked to name a single support intervention that was a priority to help them deal with the vulnerabilities associated with cattle farming. Overall, veterinary-related support interventions dominated, accounting for more than 70% of the support farmers required. The supply of drugs (41%), veterinary services (22%), and subsidies on drugs (9%) were singular priority interventions the farmers reported that they needed to reduce cattle losses. The top three support interventions required by cattle farmers in the BY district included supply of drugs (37%), provision of veterinary services (25%), and provision of water points (12%). Whereas, in the KAPS district these required support interventions were all veterinary-related: supply of drugs (45%), veterinary services (19%), and subsidies on drugs (11%); see (Figure 2). The proportions of farmers reporting support interventions preferred did not differ significantly by study district (results not shown).

The RS-14 exhibited strong psychometric properties, with Cronbach’s alphas (α) of 0.87. The study found that the resilience of the cattle farmers was relatively high with a mean resilience score of 71 (SD = 8) out of a possible 98, with farmers performing best on the perseverance sub-scale (see Table 2). The cattle farmers on average scored more than 73% of the total possible resilience scores on the dimensions of perseverance, self-reliance, authenticity, and purpose. The farmers scored an average of 69% on the equanimity dimension. Overall, more than half, 52% (150/287), of the farmers had high resilience. The farmers that are highly resilient scored on average 78 out of 98 points on the RS-14 scale (minimum score = 72 and maximum score = 91). Conversely, those with low resilience scored an average of 64 out of 98 points (minimum score = 50 and maximum score = 71).

### 3.2. Factors Influencing Resilience of Cattle Farmers

Table 3 shows the univariable linear regression results for each of the hypothesized factors and cattle farmers’ resilience scores. The livestock farmers’ resilience was significantly higher if they had experience with raising livestock from childhood. The resilience scores, although higher for cattle farmers in the KAPS district, were not significantly different from farmers in the BY district (*p* = 0.06). The association between resilience and educational attainment was mixed. Only farmers that attained tertiary education had significantly higher resilience compared to those with no formal education (*p* = 0.02).

Further, the livestock farmers’ resilience improved significantly with each additional year increase in age, each additional person in a household, and each additional cattle in a herd (*p* < 0.001). Each additional cattle and additional proportion of herd lost were also significantly associated with higher resilience scores.

To assess the determinants of high resilience levels among the livestock farmers, multivariable logistic regression analysis of the factors independently associated with resilience was performed. We included variables that were statistically significant at the 10% level in the multivariable analysis (Table 4). The proportion of the herd lost was excluded as it was derived from the total number of cattle lost and the herd size. We evaluated and found no evidence of multicollinearity in the independent predictor variables (Table S2, Supplementary Material).

In an unadjusted model, the livestock farmers’ resilience was positively and significantly influenced by the farmer’s age, the number of persons in a farmers’ household, the herd size, the lived experience with livestock rearing, and the total number of cattle lost to adverse events. Although farming in the Kwahu Afram Plains South district was associated with 4% increased odds of high resilience compared to farming in Bunkpurugu-Yunyoo (crude odds ratio (cOR) = 1.04 (95% CI = 0.66–1.67), *p* < 0.1), this difference was not statistically significant (Table 4).

Significantly, farmers who had experience with raising livestock since their childhood were three times more likely to be highly resilient compared to the farmers who did not have this experience (cOR = 3.06 (95% CI = 1.81–5.20), *p* < 0.001). Moreover, an additional year of age increases the likelihood of having high resilience by 1.05 (cOR = 1.05 (95% CI = 1.02–1.07), *p* < 0.001). An additional cattle in a farmers’ herd also significantly increases the likelihood of having high resilience by 1.02 (cOR = 1.02 (95% CI = 1.01–1.03), *p* < 0.001). As an additional cattle is lost to adverse events, the likelihood of farmers’ having high resilience increases by 1.07 (cOR = 1.06 (95% CI = 1.03–1.11), *p* < 0.001).

After adjusting for farmers’ educational attainment, household size, the district where animals are raised, and the total number of cattle lost to adverse events, the farmer’s resilience level was significantly influenced by age, herd size, and experience with raising livestock (pseudo R^2^ = 0.17, *p* < 0.001).

The cattle farmers who had experience with raising livestock during their childhood were 2.8 times more likely to be highly resilient compared to the farmers who are new to livestock farming (adjusted odds ratio (aOR) = 2.81, 95% CI = 1.57–5.02, *p* < 0.001). As farmers age, the adjusted odds of having high resilience increase by 1.04 with each additional one-year increase in age (aOR = 1.04, 95% CI = 1.01–1.06, *p* < 0.01). Similarly, the adjusted odds of being highly resilient increases by 1.02 with each additional cattle in the herd (aOR = 1.02, 95% CI = 1.01–1.03, *p* < 0.001).

## 4. Discussion

Livestock production serves as a means of livelihood to support livestock-dependent populations and contributes to the growth of local economies. In Ghana, livestock farming is raised mainly under the extensive system, where farmers move their animals to maximize on pastures from occasional rainfall [31]. Cattle are mainly raised alone (pastoral system) or in combination with growing crops (agro-pastoral system). Some farmers also combine raising cattle in addition to other ruminants like sheep and goats [32]. However, livestock farmers continue to face many adverse events including animal disease epidemics, pasture and water shortages and conflicts that negatively affect their livestock productivity and profitability. The previous work among livestock farmers in Ghana demonstrated how animal losses contribute to distressing farmers’ livelihoods and mental health [7]. The current research applied the resilience framework of Obrist and colleagues [11] to identify the vulnerabilities of farmers in different agroecological zones as well as their capacity to overcome these adversities. In many sub-Saharan African countries, cattle are kept mainly as a principal store of wealth, enabling them to be mobilized in times of crises [33]. To identify the vulnerabilities and resilience levels of households that keep cattle, this study targeted the head of households that keep cattle in two agroecological zones in Ghana.

We found that despite facing multiple adversities with consequent losses of livelihoods, more than half of the cattle farmers had high resilience, particularly if they are experienced in livestock farming and have large herd sizes. Similar findings were made in the Turkana County in Kenya, where only one-third of pastoralists were found to be highly vulnerable to climate-induced adversities including droughts, floods, animal disease outbreaks, and conflicts. Forty-four percent were found to have the capability to adapt to adversities with assistance, while twenty-nine percent of the pastoralists could cope on their own to the adversities [25]. The Intergovernmental Panel on Climate Change (IPCC) describes vulnerability as a predisposition to be negatively affected by adverse events. The IPCC notes that in relation to climate change and its effect on individuals or groups, vulnerability can be described as the susceptibility, sensitivity, and lack of resilience or capacities of the exposed person or group to cope with and adapt to extremes and non-extremes [34]. Thus, although, the authors in the study among Turkana pastoralists [25] did not directly measure resilience, they instead assessed the lack of it through vulnerability assessment based on socio-economic and biophysical positives and negatives peculiar to pastoral households. Future studies among these livestock-dependent populations could apply the RS-14 scale in their settings to directly measure resilience. The RS-14 is easily applicable in measuring individual-level resilience to adversities. The constructs evaluated: equanimity (balanced perspective of one’s life), meaningfulness (understanding that life is meaningful and valuable), perseverance (ability to keep going, even after setbacks), self-reliance (belief in one’s abilities and awareness of limitations). and existential aloneness (the recognition of one’s unique path and acceptance of one’s life) [27] are particularly relevant for the peculiar circumstances of livestock-dependent populations. The RS-14 has been applied in young individuals with different psychosocial circumstances (adolescents and young adults with or without special social problems) and has demonstrated consistent cohesiveness in distinguishing between different constructs across groups. It showed a positive correlation with adaptive concepts and negative correlation with psychological distress in the groups with and without special problems [35]. Similar findings have been made with the RS-14 in other contexts [36,37,38,39]. Thus, overall, it has demonstrated good construct validity to be used in resilience assessment, whereby highly resilient individuals exhibit a significant positive relationship with adaptive concepts and vice versa.

Typically, recurrent exposure to adversity makes one robust in the face of commonly-face difficulties, as previous studies have shown in Africa [12,16,40]. Animal disease outbreaks continue to rank high as one of the main sources of livestock mortality in sub-Saharan Africa. In East Africa, for instance, several episodes of Rift Valley Fever, mange, peste des petits ruminants, contagious caprine pleuropneumonia, contagious bovine pleuropneumonia, and foot and mouth disease, have caused high livestock mortalities and production shortfalls [41,42,43,44]. Similar ravaging effects of infectious diseases have been reported in the West and Central African regions [7,45,46]. Other adversities like droughts and pasture shortages, conflicts, and theft of animals have also been reported in some parts of the continent [25,47,48,49]. However, as our results showed, the vulnerabilities affecting livestock production appear to contribute to the farmers’ capacity to adapt and overcome adversity. However, when other predictors of resilience are included in the model, the apparent effect of the number of animals lost on resilience wanes (See Table 4). The design of our study, however, does not permit us to determine the temporal relationship between resilience levels and the number of cattle lost to adverse events. It is plausible that farmers could learn from adversities already faced to plan and respond better to future adversities.

Livestock farmers adapt to adversities in different ways, some being positive and others not. In some areas where animal disease outbreaks affect livestock farms, they tend to resort to self-treating of animal diseases [42,50]. In the previous work [7], livestock farmers in Ghana similarly adopted the strategy of treating animal diseases themselves without veterinary advice and often using drugs that are not properly regulated. Another key threat to public health was the sale of diseased animals to recover some losses. These adaptive strategies cannot be sustainable and hence require optimal interventions that would repurpose livestock farmers’ industry. We found it intuitive that as farmers’ herd sizes increase, they tend to be more resilient, showing that increased livestock population can be the main strategy of an intervention if implemented complying to the availability of resources i.e., feed for the livestock and credits for farmers to invest in better animal health. One approach, therefore, will be an intervention designed to improve the productivity of the local breeds as well as preventive veterinary services like vaccinations to reduce animal disease outbreaks. These strategies could help farmers to maintain highly productive herds, thereby contributing to improved wellbeing and food security. Further research that evaluates livestock farmers’ valuation and preference for vaccines will help inform preventive veterinary interventions. This notwithstanding, some adaptive strategies utilized by livestock farmers, like the growing of crops and the raising of mixed livestock species, are good strategies that would promote the profitability of farmers and public health in general [44,51]. It was found that agro-pastoralists in our study were less resilient than those keeping cattle alone. This phenomenon may be explained by cumulative adversities from both crop and livestock farming ventures of agro-pastoralists, thereby overwhelming them. It is worth noting that poor access to finance services and over-reliance on rain-fed farming remain key weaknesses affecting crop farmers in Ghana especially [52].

Although improved access to support interventions that target vulnerabilities enhances resilience [21], cattle farmers do not appear to have this support system. About one-third of the farmers in the current study reported receiving no support interventions to help them deal with the vulnerabilities associated with cattle farming. Even for those who received some support, mainly from veterinary services and/or family members, this support did not appear sufficient to deal with what the farmers faced. This is explained by almost two-thirds of the farmers in this study still requiring veterinary-related interventions to help them deal with animal diseases, even though the majority of the reported support received by the farmers was veterinary-related. As previous studies have shown, the support interventions that could cushion livestock farmers towards sustainability on the continent remain woefully inadequate due to underinvestment and sometimes neglect of the sector [53,54]. Thus, although resilience levels improved for each additional support received, the impact remained non-significant. Further studies are needed to determine the performance of veterinary services and interventions targeting pastoral and agro-pastoral specific needs, to guide policy decisions for the livestock sector in Ghana.

Given the growing perception and evidence of the tendency of especially mobile pastoralists to be involved in violence and conflicts, mostly derived from the vulnerabilities they face in some sub-Saharan African countries including Ghana [5,55,56], the provision of support interventions could be preventive. These support interventions could promote the adoption of sustainable resilience strategies that can eschew violent tendencies among these livestock-dependent populations. In addition, the increased stigmatization of an already vulnerable group could worsen the choices they make by driving some of them to join violent, or worse, terrorist groups, as observations from Mali and Burkina Faso in West Africa portray [56], with devastating consequences for public security and food security.

With the current focus and drive towards enhancing sustainability in agricultural production, having highly resilient farmers is key to promote the adoption and use of agricultural innovations [34,57]. Different sustainable strategies have been implemented in anticipation of adversities, in order to prevent or enable better responses to them, when populations are resilient including the use of green belts, reduced use of chemical fertilizers, and other new technologies [34]. Large-scale sustainable livestock production in advanced nations has relied on the adoption of new innovations that promote livestock productivity while reducing the effect on the environment [58,59]. However, the uptake of technologies that could promote sustainable livestock agriculture such as improved breeds, feeding, vaccination, and milk automation, among others, is slow in developing countries [60]. Many factors contribute to this low phenomenon, including failure of engagement of end-users [61], poverty [62], lack of awareness and inappropriateness of practices [63], and lack of suppliers of new technological inputs and services [60]. The high prevalence of infectious diseases due to a lack of sustained implementation of control measures also discourages adoption of exotic breeds, which may be less tolerant to these diseases [64]. Furthermore, marginalized populations usually lack the economic and political power to shape innovations to meet their needs [65]. Therefore, by applying the One Health approach, which emphasizes transdisciplinary discourse, marginalized and vulnerable populations like livestock producers can make meaningful contributions to innovations that seek to improve their livelihoods. If these reforms are made to the innovation process through the enhancement of co-designing and co-innovation, new technologies can be adapted better to farmers’ needs and would promote ownership and adoption [61,65].

Few studies employed a direct quantitative assessment of the resilience levels of agriculturalists including crop farmers and livestock-dependent populations in sub-Saharan Africa. Previous studies either target vulnerabilities or adaptive strategies of farmers to climate-induced adversities [25,66,67,68,69,70]. Our study provides an approach to directly assess the resilience levels of farmers, especially vulnerable livestock-dependent populations. In spite of this, our final adjusted model accounted for only a quarter of the variations observed in farmers’ resilience. This may be due to our focus on farming-related determinants. Further studies could assess other factors in farmers’ lives that could contribute to their resilience to adverse events affecting the raising of livestock. However, our study had some limitations. The unavailability of formal census data on livestock and farmers’ population at the time of the study did not permit the use of randomization techniques in recruiting study respondents. We believe that the recently conducted livestock census, when published, would provide crucial data about the sector to improve sampling in future surveys.

## 5. Conclusions

The occurrence of animal diseases, droughts, and conflicts, among other adverse events, threatens livestock productivity, leading to heightened food insecurity and vulnerability of farmers to poverty. Despite these adversities confronting them, the livestock farmers had relatively high resilience, coping with the shocks and adopting strategies that enable them to anticipate and minimize their impact in the longer term. Therefore, there remains an opportunity for key policy makers to harness this resilient capacity of farmers for supporting food security and health system strategies. Farmers who had experience with raising livestock during childhood tended to be more resilient as did those with larger herds. The implementation of interventions that include peer-training for farmers without prior experience in livestock farming before they venture into livestock farming could help them become more resilient. Additionally, strategies must be adopted to mitigate the impact of infectious diseases on livestock production, through improving access to financial resources and quality veterinary services. This will foster sustainable improvements in herd population and productivity and farmers’ wellbeing. Research should evaluate farmers preferences for the livestock system transition as far as climate and emerging markets are concerned. Further studies are also needed to determine the performance of veterinary services and interventions targeting pastoral- and agro-pastoral-specific needs to improve prosperity in the sector.

## Figures and Tables

**Table 1:** Resilience scale (RS-14) and the resilience characteristics (facet) measured.

Resilience Facet	Questions	Strongly Disagree				Strongly Agree		
Self-reliance	I usually manage one way or another	1	2	3	4	5	6	7
	I feel that I can handle many things at a time	1	2	3	4	5	6	7
	I can get through difficult times because I have experienced difficulty before	1	2	3	4	5	6	7
	In an emergency, I am someone people can generally rely on	1	2	3	4	5	6	7
	When I am in a difficult situation, I can usually find my way out of it	1	2	3	4	5	6	7
Purpose	I feel proud that I have accomplished things in my life	1	2	3	4	5	6	7
	I keep interested in things	1	2	3	4	5	6	7
	My life has meaning	1	2	3	4	5	6	7
Equanimity	I usually take things in stride	1	2	3	4	5	6	7
	I can usually find something to laugh about	1	2	3	4	5	6	7
Perseverance	I am determined	1	2	3	4	5	6	7
	Self-discipline is important	1	2	3	4	5	6	7
Authenticity	I am friends with myself	1	2	3	4	5	6	7
	My belief in myself gets me through hard times	1	2	3	4	5	6	7

**Table 2:** Summary of resilience and its dimension scores for cattle farmers in the Bunkpurugu-Yunyoo (BY) and Kwahu Afram Plains South (KAPS) districts.

Variable	Number of Items	Mean (SD)—BY	Mean (SD)—KAPS	*p*-Value
Resilience	14	70.3 (8.4)	72.0 (7.8)	0.064
Self-reliance	5	25.5 (3.4)	25.8 (3.2)	0.387
Purpose	3	14.9 (1.9)	15.3 (2.0)	0.117
Equanimity	2	9.7 (1.5)	9.5 (1.6)	0.269
Perseverance	2	10.3 (1.5)	11.0 (1.5)	<0.001
Authenticity	2	9.9 (1.5)	10.5 (1.6)	<0.001

Means (SD) are the arithmetic mean and their respective standard deviations in parentheses for farmers’ scores on the resilience scale dimensions in each study district. *p*-values denote results from a Student t-test analysis of equal means between the study districts.

**Table 3:** Univariable analysis of resilience levels and farmer characteristics.

	Resilience		
Variable	Slope	95% (CI)	*p*-Value
Sex of farmer			
Female	ref		
Male	−1.82	(−5.63, 1.99)	0.348
Highest level of education			
No formal education	ref		
Basic education	1.13	(−0.99, 3.24)	0.295
Secondary school education	−2.77	(−5.61, 0.07)	0.056
Tertiary education	5.49	(0.99, 9.99)	0.017
Marital status			
Not married	ref		
Married	−1.67	(−5.23, 1.89)	0.358
District			
Bunkpurugu-Yunyoo	ref		
Kwahu Afram Plains South	1.78	(−0.11, 3.67)	0.064
Age of farmer	0.22	(0.14, 0.30)	<0.001
Number in household	0.42	(0.24, 0.60)	<0.001
Number of cattle in the herd	0.05	(0.03, 0.07)	<0.001
Childhood experience with raising livestock			
No	ref		
Yes	4.53	(2.53, 6.52)	<0.001
Farmer type			
Pastoralist	ref		
Agro-pastoralist	−2.55	(−5.84, 0.74)	0.128
Number of adverse events experienced	0.04	(−0.51, 0.60)	0.874
Total number of cattle lost	0.26	(0.15, 0.37)	<0.001
Proportion of cattle lost	0.09	(0.02, 0.16)	0.008
Number of support interventions received	0.03	(−0.54, 0.60)	0.918

“Slope” denotes coefficients that are unstandardized linear regression slopes with 95% confidence intervals (95% CI) in parentheses and corresponding *p*-values. “Variable” include hypothesized factors to influence farmers’ resilience. “Not married” comprises farmers who are either single, widowed, or divorced. “ref” denotes the reference category for nominal variables.

**Table 4:** Factors predicting high resilience levels of cattle farmers in the Bunkpurugu-Yunyoo and Kwahu Afram Plains South districts, Ghana.

	Unadjusted Model		Adjusted Model	
Variables	cOR (95% CI)	*p*-Value	aOR (95% CI)	*p*-Value
Age of farmer	1.05 (1.02, 1.07)	<0.001	1.04 (1.01, 1.06)	0.007
Number in household	1.11 (1.05, 1.17)	<0.001	1.05 (0.99, 1.12)	0.134
Number of cattle in the herd	1.02 (1.01, 1.03)	<0.001	1.02 (1.01, 1.03)	<0.001
Total number of cattle lost	1.07 (1.03, 1.11)	<0.001	1.01 (0.96, 1.05)	0.749
Highest level of education				
No formal education	ref		ref	
Basic education	1.07 (0.63, 1.81)	0.799	1.28 (0.71, 2.31)	0.415
Secondary school education	0.61 (0.30, 1.26)	0.182	0.79 (0.35, 1.77)	0.565
Tertiary education	3.37 (0.89, 12.9)	0.075	3.74 (0.83, 16.8)	0.086
Experience with raising livestock				
No	ref		ref	
Yes	3.06 (1.81, 5.20)	<0.001	2.81 (1.57, 5.02)	<0.001
District				
Bunkpurugu-Yunyoo	ref		ref	
Kwahu Afram Plains South	1.04 (0.66, 1.66)	0.853	0.78 (0.44, 1.38)	0.395

Variables were included as predictors of cattle farmers’ resilience if they were statistically significant at the 10% level in the univariable analysis, except for the proportion of the herd lost, which was excluded (Table 2 above). Crude odds ratio (cOR) with a 95% CI and the associated *p*-value for the unadjusted model and adjusted odds ratio (aOR) with a 95% CI and the associated *p*-value for the adjusted model. “ref” denotes the reference category for nominal variables.

## Data Availability

All data generated or analyzed during this study are included in this published article and its supplementary information files.

## Supplementary Materials

The following are available online at https://www.mdpi.com/article/10.3390/ijerph18179008/s1, Table S1: Sampling frame of farming communities, Table S2: Variance Inflation Factors (VIF) of independent predictor variables.
