# Supplementary material for: Coping with Adversity: Resilience Dynamics of Livestock Farmers in Two Agroecological Zones of Ghana
Source: Int J Environ Res Public Health. 2021 Aug 26;18(17):9008. doi: 10.3390/ijerph18179008 (PMC8430896; doi:10.3390/ijerph18179008)
Supplement: Supplementary file 1 [file ijerph-18-09008-s001.zip › Table S1.pdf]

SAMPLING FRAME OF FARMING COMMUNITIES WITH CATTLE FARMERS IN THE KWAHU AFRAM PLAINS SOUTH DISTRICT

1. AGYATA
2. AMANHYIA
3. AMEDZOFE
4. AMEYAW
5. ATAKORA
6. ATONSU
7. BATOR-KOFE
8. BONKPATA
9. DEDESO
10. DIM SAKABO
11. DOMEABRA
12. DOTORPONG
13. EKYE
14. FASO-KRACHI
15. FORIFORI
16. FOSU-TOMEFA
17. GADORKOPE
18. GODODONUKOPE
19. GODOPE
20. GODZIKOFE
21. HLIHADZI
22. KOMLA-KWAO
23. KWAME DWAMENA
24. KWASI-ADAE
25. KWASIKUMAH
26. MAFIKOPE
27. MRANDAN
28. NEKPOKOPE
29. NYAFRED
30. ODOTOMU
31. ODUMASUA
32. OFFINSO
33. OGBODOKOPE
34. SUMSE
35. SEMENHYIA
36. TEASE
37. TRIBU
38. TWENFOUR FASO
39. WAWASE
40. XEDZODZOE-KOPE

SAMPLING FRAME OF FARMING COMMUNITIES WITH CATTLE FARMERS IN THE BUNKPURUGU-YUNYOO DISTRICT

1. BAMANGO
2. BILFACO POIGA
3. BINDE
4. BOATERIGU
5. BUMBONG
6. BUNBUNA
7. BUNKPURUGU ZONGO
8. CHINTULUNG
9. DAGBAN
10. DALOUR
11. GBANKONI
12. GBANKURUGU
13. GBETMUNPAK
14. GBINGBANI
15. GOMSUKA
16. GUAGBIANG
17. JAGOUK
18. JANDERI
19. JILIK
20. JIMBALE
21. KAMBA KONKOOK
22. KAUK
23. KINKANGU
24. KPAGYALA
25. KPANLORI
26. KPEKPALGBENI
27. KPEMALE
28. KPENTAUNGKUNKWADAN
29. KUNPAK TAMBING
30. MAJIA
31. MANGOR
32. NAABAUK
33. NABULIK
34. NAJONG
35. NAKPANDURI
36. NAMANGAI
37. NAMGBAM
38. NAMPOUTIBAUK
39. NANGOBIK
40. NANYIAR
41. NASUAN
42. PAGNATIK
43. PULI
44. SAKBOUK
45. SANJAAK
46. TABOKURUGU
47. TATARA
48. TIENKINLU

49. TINKPANG
50. TOJING
51. TUNA
52. TUSUGU
53. WAWA
54. YUNYOO

List of communities and number of livestock farmers sampled

District	Community	Number of farmers sampled
Bunkpurugu-Yunyoo	Bamango	12
	Bunkpurugu Zongo	11
	Gbankurugu	13
	Gbetmunpak	14
	Jagouk	12
	Kauk	13
	Kinkangu	12
	Naabauk	14
	Pagnatik	10
	Tatara	13
	Tojing	10
	Yunyoo	11
Kwahu Afram Plains South	Agyata	13
	Dim Sakabo	14
	Dotopong	13
	Ekye Amanfrom	17
	Forifori	17
	Fosu	16
	Gadorkorpe	15
	Kwame Dwamena	13
	Kwasi Addae	11
	Tease	13
