# Supplementary material for: Coping with Adversity: Resilience Dynamics of Livestock Farmers in Two Agroecological Zones of Ghana
Source: Int J Environ Res Public Health. 2021 Aug 26;18(17):9008. doi: 10.3390/ijerph18179008 (PMC8430896; doi:10.3390/ijerph18179008)
Supplement: Supplementary file 1 [file ijerph-18-09008-s001.zip › Table S2.pdf]

Variance Inflation Factors (VIF) of independent predictor variables

**Parameter estimates**				
**Term**	**Estimate**	**Standard error**	***p*-value**	**VIF**
**Age of farmer**	0.152	0.04	<0.001	1.21
**Number in household**	0.165	0.09	0.082	1.21
**Number of cattle in the herd**	0.027	0.01	0.025	1.37
**Total number of cattle lost**	0.055	0.06	0.388	1.53
**Highest level of education**				
Basic education	1.645	0.98	0.095	1.31
Secondary education	-0.977	1.35	0.471	1.33
Tertiary education	5.664	2.13	0.008	1.16
**Experience with raising livestock**				
Yes	3.545	0.95	<0.001	1.05
**District**				
Kwahu Afram Plains South	0.739	0.94	0.432	1.21

F(9, 277) = 9.62

R-squared = 0.24

Prob > F = <0.001

Mean VIF = 1.27
